# Prospective longitudinal study of frailty transitions in a community-dwelling cohort of older adults with cognitive impairment

**DOI:** 10.1186/s12877-015-0174-1

**Published:** 2015-12-29

**Authors:** Mei Sian Chong, Laura Tay, Mark Chan, Wee Shiong Lim, Ruijing Ye, Eng King Tan, Yew Yoong Ding

**Affiliations:** Institute of Geriatrics and Active Ageing, Tan Tock Seng Hospital, 11 Jalan Tan Tock Seng, S30843 Singapore, Singapore; Department of Geriatric Medicine, Tan Tock Seng Hospital, 11 Jalan Tan Tock Seng, S308433 Singapore, Singapore; Department of Neurology, Singapore General Hospital, Outram Rd, S169608 Singapore, Singapore; Duke-NUS Graduate Medical School, 8 College Road, S169857 Singapore, Singapore

**Keywords:** Frailty, Transitions, Cognitive impairment

## Abstract

**Background:**

Frailty and cognitive impairment are seemingly distinct syndromes, but have a shared vulnerability to stress in older adults, resulting in poorer outcomes. Although there has been recent interest in cognitive frailty, frailty transitions in relation to cognitive deterioration in older adults with cognitive impairment have not yet been well studied. We thus aim to study frailty transitions and change in cognitive status over 1-year follow-up among subjects with cognitive impairment attending a tertiary Memory Clinic.

**Methods:**

This is a prospective cohort study of mild cognitive impairment (MCI) and mild-moderate Alzheimer’s disease (AD) community-dwelling subjects. We obtained data on clinical measures, muscle mass and physical performance measures. Cognitive status was measured using Chinese Mini-Mental State Examination (CMMSE) and Clinical Dementia Rating-Sum of Boxes (CDR-SB) scores. We measured gait speed, hand grip strength, exhaustion and weight loss at baseline, 6 and 12 months to classify subjects according to the modified Fried criteria (involving strength, gait speed, body composition and fatigue) into non-frail (<2 frail categories) and frail categories (≥2 frail categories). Frailty transitions between baseline and 12-months were assessed. We performed random effects statistical modelling to ascertain baseline predictors of longitudinal frailty scores for all subjects and within MCI subgroup.

**Results:**

Among 122 subjects comprising 41 MCI, 67 mild and 14 moderate AD, 43.9, 35.8 and 57.1 % were frail at baseline respectively. Frailty status regressed in 32.0 %, remained unchanged in 36.0 %, and progressed in 32.0 % at 12 months. Random effects modelling on whole group showed longitudinal CDR-SB scores (coeff 0.09, 95 % confidence interval (CI) 0.03–0.15) and age (coeff 0.04, 95 % CI 0.02–0.07) to be significantly associated with longitudinal frailty score. Among MCI subjects, only female gender (coeff 1.28, 95 % CI 0.21–2.36) was associated with longitudinal frailty score, while mild-moderate AD subjects showed similar results as those of the whole group.

**Conclusions:**

This is the first study to show longitudinal frailty state transitions in cognitively-impaired older adults. Frailty transitions appear to be independent of progression in cognitive status in earliest stages of cognitive impairment, while mild-moderate AD subjects showed associations with age and cognitive deterioration. The potential for cognitive frailty as a separate therapeutic entity for future physical frailty prevention requires further research with a suitably powered study over a longer follow-up period.

## Background

Research efforts over the past decade have focused on defining the clinical and physiological characteristics of frailty and its relationship to adverse health outcomes [1, 2]. The relative lack of concrete data on the relationship between cognition and neurodegeneration in mediating frailty progression, has fuelled calls for more research into this area [3]. Recent research has focused on the proposed entity of “cognitive frailty” [4, 5] to describe a clinical condition that is characterized by simultaneous occurrence of physical frailty and cognitive impairment in the absence of overt dementia. This potentially could be a preventive or therapeutic target to prevent both cognitive and functional decline, and frailty progression.

Many cross-sectional studies demonstrated the relationship between general cognitive function and physical frailty [6], although less consistently with dementia [7]. Sub-domains of gait speed and grip strength appear to be more strongly associated with cognitive function, in contrast to the association of executive function with frailty [6, 8]. Additionally, Alzheimer’s disease (AD) pathology is associated with physical frailty [9]. Conversely, physical frailty has been associated with incident mild cognitive impairment (MCI) and Alzheimer’s dementia, and greater rate of cognitive decline in older persons [10, 11]. The surge of transition studies in recent years have mainly focused on physical frailty [12–15], with a relative paucity of data available for concomitant transitions in cognitive status. A recent cross-sectional study suggests a U-shaped relationship between frailty and cognition, characterized by initial dissociation with cognitive impairment and subsequent convergence at later stages [16].

Frailty among older persons have been shown to be a dynamic process, characterized by frequent transitions between frailty states over time [15, 17]. Despite the interest in the area, most of the studies have looked at transition in frailty and health status using multistate modelling methods employing deficit counts [14, 18, 19]. More recent studies have highlighted age, medical factors [12, 20] and higher socioeconomic status to be protective [12]. Only one published study has looked at baseline cognition scores and frailty to be strongly associated with changes in cognitive status over time in the Canadian Study of Health and Aging (CSHA) study [21].

Functional deterioration constitutes the most significant contributor to informal and formal costs of dementia care [22]. Despite recent advances in understanding the pathophysiology of AD and numerous drug trials, effective interventions to prevent or delay functional decline in dementia remain elusive It has been suggested that cognitive frailty might represent the earliest spectrum of the clinical entity [4, 5]. Because frailty represents a separate therapeutic target for dementia subjects, cognitive frailty may thus present a potential window of opportunity for targeted interventions to delay overt functional decline and disability. A deeper understanding of the trajectory and predictors of frailty transitions across the different stages of AD could allow further investigation into different stage-specific pathogenic mechanisms akin to the different biomarker stages in AD pathology [23], thus allowing for targeted interventions to delay functional decline and disability.

Thus, in this prospective study of community-dwelling older adults with different stages of cognitive impairment from MCI through to mild-moderate AD stages, we aim to study the relationship between frailty transitions and change in cognitive status over one year.

## Methods

This is a prospective cohort study of MCI and mild-moderate probable AD community-dwelling subjects attending a specialist Memory Clinic, Cognition and Memory Disorders Service, Tan Tock Seng Hospital, Singapore between the period of December 2010 to December 2014. Ethics approval was obtained from the Domain Specific Review Board (DSRB) of the National Healthcare Group (NHG). Written consent was obtained from the patient or legally acceptable representative where appropriate.

### Study groups

MCI was defined as: (a) global Clinical Dementia Rating (CDR) [24] score of 0.5; (b) subjective memory complaint which is corroborated by a reliable informant; (c) delayed recall >1 SD below age and education-adjusted means derived from an earlier normative study [25]; (d) relatively normal general cognitive function (Chinese Mini Mental State Examination (CMMSE) [26] score ≥ 21 and ≥ 24 for subjects with ≤6 and >6 years education respectively; and (e) largely intact activities of daily living; and [6] no clinical dementia.

Mild-moderate AD subjects fulfill diagnoses of probable AD using the National Institute of Neurological and Communicative Disorders and Stroke and Alzheimer’s Disease and Related Disorders Association **(**NINCDS-ADRDA) criteria) [27] and had a global CDR score of 0.5–2.0 respectively. We excluded subjects with a diagnosis of possible AD.

### Inclusion and exclusion criteria

Subjects are eligible if they fulfilled the following: age ≥ 55 years; diagnosis of MCI or mild to moderate AD; availability of a reliable informant; and community dwelling. We excluded subjects with presence of other central nervous conditions (stroke disease, Parkinson’s disease, subdural hematoma, normal pressure hydrocephalus, and brain tumor), presence of systemic conditions that can contribute to CI (hypothyroidism, B12 deficiency, and hypercalcaemia), presence of any active neuropsychiatric conditions producing disability, and residence in a sheltered or nursing home. Grip strength exclusions included recent pain in the wrist or hand or a history of surgery on the upper extremity in the 3 months preceding assessment.

The validity of the overall cognitive evaluation process and CDR scoring has been previously established [24, 28]. Laboratory investigations excluding potentially reversible causes of dementia via blood tests and neuroimaging were done. A multidisciplinary consensus meeting was conducted to review all relevant results for accurate clinical phenotyping. Patients meeting study eligibility were then included in the study.

### Baseline data collection

#### Demographics, cognitive and functional assessment

We collected data on: (a) baseline demographics (age, gender, years of education); (b) general cognitive function (CMMSE); (c) dementia severity (CDR global and sum-of-boxes (CDS-SB) score) [24]; (d) physical functioning (Barthel’s basic activities of daily living (ADL) index [29] and Lawton’s instrumental ADL (iADL) index [30]); and (e) behavioral symptoms using the Neuropsychiatric Inventory (NPI) [31] and the Diagnostic and Statistical Manual, 4th ed (DSM-IV) for Major Depression [32]. MCI subjects also underwent a neuropsychological assessment that evaluated amnestic (immediate, delayed and recognition verbal memory) and non-amnestic (language, attention, executive function and visuospatial function) domains [23].

#### Vascular risk factor profile

Data on hypertension, hyperlipidemia, diabetes mellitus, atrial fibrillation, peripheral vascular disease, smoking history, ischemic heart disease, and body mass index were collected. Multi-morbidity was calculated based on summation of the above factors (excluding BMI) (maximum score of 7).

#### Neuroimaging

Brain computed tomography (CT) scan or magnetic resonance imaging (MRI) was performed. White matter lesions (WML) severity was graded using the Age-Related White Matter Changes (ARWMC) scale by a blinded rater (L.T.) [33]. Medial temporal atrophy (MTA) score (reflecting neurodegeneration) was scored on T1-weighted coronal slices parallel to the brainstem axis and perpendicular to the hippocampal axis, by a consensus method where the scores range from 0 (no atrophy) to 4 (severe atrophy) [34].

#### Frailty and related nutritional and activity measurements

A single blinded assessor (Y.R) measured the four components of grip strength, timed walk, unintentional weight loss (>3 kg) and fatigue to yield a modified Fried criteria [1], which is better operationalized in the clinical setting. The frailty scoring was done by a separate assessor (CMS). Grip strength was measured using the hydraulic hand dynamometer (North Coast@ Hydraulic Hand Dynamometer). Two trials of grip strength were obtained for each hand with all 4 trials averaged to yield a final strength score. Gait speed was based on the time taken to walk 15 ft (4.5 m). Published Asian cut-offs were used to define each frailty sub-item (grip strength <26 kg for males and <18 kg for females scored as 1; and gait speed <0.8 m/s scored as 1) [35]. Lastly, we modified 2 questions from the Center for Epidemiologic Studies-Depression Scale to assess fatigue. Participants answered yes/no to the following: (a) I felt that everything I did was an effort, and (b) I could not get “going”. The frailty sub-item of fatigue was considered positive if either question was endorsed. A subject was classified as frail if the summated sub-item scores were ≥ 2 and non-frail if <2. The rationale for the 4 categories and exclusion of physical activity was based on Buchmann data [10] for AD. Physical activity of subjects were obtained by way of Frenchay activity scale [36].

Nutrition was assessed using the Mini Nutritional Assessment (MNA) questionnaire [37].

#### Muscle mass measurements

Muscle mass (lean and fat mass of upper and lower extremity and percentage body fat) was measured via Dual energy X-ray absorptiometry (DXA) Hologic machine Discovery Series APEX 13.3-Model**.** DXA currently represents the more accessible technique for body composition assessment. It accurately provides estimates of lean, fat, and bone tissues in the entire body or in specific regions. The coefficient of variation (CV) is 0.39 %. Appendicular Skeletal mass (ASM)/ht^2^ was calculated by the summation of muscle mass measured in the four limbs divided by square of height (in metres). The Short Physical Performance Battery (SPPB) [38] was used to measure physical performance.

#### Vitamin D, lipid status and APOE genotyping

We measured total 25-hydroxy vitamin D level with the CV at 17.8ug/L was 7.2 %. Fasting total cholesterol, high-density lipoprotein (HDL) level, low-density lipoprotein (LDL) and triglyceride (TG) levels were obtained from the participants. Apolipoprotein E (APOEε4) is a plasma cholesterol transport molecule which has been postulated to be associated with AD. Importantly, APOEε4 status may exert a modulatory effect on disease trajectory and clinical expression of disease. We performed APOE genotyping into APOEε2, 3, 4 isoforms via restriction enzyme analysis using applied biosystems platform- ABI Prism 310 Genetic Analyser.

### 6 and 12-month data collection

Frailty and cognitive measures were collected at baseline, 6 and 12-month follow-up period.

Frailty transitions were defined based on differences in frailty scores between baseline and 12 months, with decrease (≥ −1 point), unchanged (0 point) or increase (≥1 point) corresponding to regressed, unchanged and progressed frailty states respectively.

### Statistical analyses

We performed univariate analysis to examine differences in baseline characteristics between the 3 cognitive subgroups (MCI, mild AD and moderate AD), using analysis of variance (ANOVA) with Bonferroni correction for parametric continuous variables (as confirmed by normality testing) and Kruskal-Wallis test for nonparametric continuous variables. Chi-square test was performed for categorical variables among the 3 cognitive subgroups. Similarly, we performed univariate analyses to compare baseline characteristics among the three frailty transition subgroups (regressed, unchanged, and progressed).

We performed random effects modelling with longitudinal frailty score as the dependent variable. We included a-priori defined covariates-including age, gender, cognition-related, functional measures, biochemical and lifestyle factors for the frailty outcome. We subsequently repeated analyses separately for MCI subjects and mild-moderate AD subjects.

Statistical analyses was performed on STATA 13.0 statistical software and statistical significance taken to be *p* < 0.05.

## Results

Among 163 eligible subjects, 41 declined to participate in the study (10 MCI and 31 mild AD subjects). Thus, there were 122 subjects in our final sample, comprising 41 MCI, 67 mild AD and 14 moderate AD. The reasons given were refusal by their caregiver or legally acceptable representative and concerns regarding blood draw. There were no age or gender differences between subjects who accepted or decline participation in the study.

Of the 122 patients, the mean age was 75.4 ± 7.2 years and 40.6 % were male (Table 1). They were predominantly of Chinese ethnicity with mean education of 7.1 ± 5.0 years.

**Table 1:** Patient baseline demographics, vascular risk profile, cognitive and functional status among 3 cognitive subgroups (MCI, mild AD and moderate AD)

	Total (*n* = 122)	MCI (*n* = 41)	Mild AD (*n* = 67)	Moderate AD (*n* = 14)
Demographics				
Age	75.4 (7.2)	72.5 (7.1)	76.3 (6.9)	79.4 (6.1)
Gender (Male %)	40.6	56.1	34.3	21.4 *
Race (Chinese %)	94.3	92.6	94.0	100
Education years	7.1 (5.0)	9.9 (4.7)	5.8 (4.6)	4.9 (4.5)
CDR global	0.9 (0.5)	0.5 (0.1)	0.9 (0.3)	1.82 (0.5)*^a,b,c^
CDR Sum of boxes	4.0 (3.0)	1.1 (0.6)	4.7 (1.8)	9.2 (2.7)*^a,b,c^
CMMSE	20.1 (5.7)	25.2 (2.3)	18.4 (4.6)	13.4 (5.4)*^a,b,c^
Medical comorbidities				
HT (%)	65.5	58.5	68.7	71.4
DM (%)	29.5	24.4	35.8	14.3
Hyperlipidemia (%)	70.5	63.4	74.6	71.4
IHD (%)	21.3	26.8	17.9	21.4
Smoker/ex-heavy smoker (%)	15.5	19.4	13.4	14.2 *
AF (%)	3.3	2.4	4.5	0
PVD (%)	0.8	2.4	0	0
Multimorbidity score	2.1 (1.2)	2.0 (1.3)	2.1 (1.2)	1.9 (1.3)
BMI	22.9 (3.2)	23.1 (2.6)	22.8 (3.6)	22.8 (2.4)
ETOH ingestion (%)	12.2	18.2	10.4	7.1
Past psy problem (%)	4.9	9.8	1.5	7.1
MDD (%)	2.5	4.9	1.5	0
Neurodegeneration and wm scores				
APOE status				
E2/e2	0.8	0	1.5	0
E2/e3	12.3	17.1	7.5	21.4
E2/e4	1.6	2.4	1.5	0
E3/e3	47.5	46.3	52.2	28.6
E3/e4	32.8	29.3	31.3	50.0
E4/e4	4.9	4.9	6.0	0
MTA score	1.2 (1.0) (*n* = 106)	0.6 (0.8) (*n* = 38)	1.5 (1.0) (*n* = 59)	1.6 (1.0) (*n* = 9)
ARWMC score	4.9 (4.2) (*n* = 117)	4.2 (3.3) (*n* = 38)	5.0 (4.8) (*n* = 65)	6.4 (3.6) (*n* = 14)
Function, behaviour and burden scores				
ADL	97.8 (5.7)	99.1 (1.9)	97.8 (5.5)	93.9 (11.1)*^a,b^
iADL	15.7 (5.4)	19.7 (3.7)	14.4 (4.7)	10.4 (4.7)*^a,b,c^
NPI total severity	2.1 (1.7)	1.4 (1.3)	2.3 (1.8)	2.9 (1.7)*^b,c^
NPI CG distress	3.6 (5.3)	1.4 (2.6)	4.1 (5.6)	7.2 (6.7)*^b,c^
Zarit total	22.6 (16.1)	20.3 (15.7)	21.7 (14.7)	29.6 (21.5)
FAI total	22.9 (8.1)	28.2 (6.9)	21.6 (6.6)	13.8 (6.9)*^a,b,c^
Nutrition, lean mass and functional performance				
MNA total	13.0 (1.5)	13.2 (1.6)	13.0 (1.4)	12.7 (1.7)
Grip strength	19.5 (7.5)	22.7 (8.2)	18.3 (6.8)	16.3 (5.9)*^b,c^
SPPB total	8.7 (2.3)	9.7 (2.3)	8.9 (2.0)	6.5 (2.4)*^b,c^
Lean mass measurement (ALM/ht2)	6.6 (8.2)	6.2 (0.9)	6.9 (9.8)	5.5 (0.7)

*Abbreviations*: *MCI* mild cognitive impairment, *AD* Alzheimer’s disease, *CDR*, clinical dementia rating, *CMMSE* Chinese mini mental state examination (range 0–28), *HT* hypertension, *DM* diabetes mellitus, *IHD* ischaemic heart disease, *AF* atrial fibrillation, *PVD* peripheral vascular disease, *BMI*, body-mass index, *ETOH* ethanol, *MDD* diagnostic statistical manual definition of major depressive disorder, *MTA* medial temporal atrophy, *ARWMC* age-related white matter changes, *ADL* activities of daily living (range 0–100), *CG* caregiver, activities of daily living (range 0–23), *NPI* neuropsychiatric Inventory, *FAI* frenchay activity index, *MNA* mini-nutritional assessment, *SPPB* short physical performance battery, *ALM/ht2* appendicular lean mass/ (height in metres) 2

*p < 0.05 between the 3 cognitive subgroups (ANOVA with Bonferroni correction for parametric and Kruskal-Wallis test for nonparametric continuous variables; Chi-square test for categorical variables)

^a^differences between mild and moderate AD

^b^differences between MCI and mild AD

^c^differences between MCI and moderate AD

### Comparison of demographics, vascular risk profile, cognitive and functional status between 3 cognitive subgroups

There were significant differences between the cognitive subgroups in terms of female preponderance 56.1, 34.3 and 21.4 % male, *p* < 0.05) and smoking history (19.4, 13.4 and 14.2 %, *p* < 0.05) between MCI, mild and moderate AD subjects respectively. As expected, cognitive and functional performance (in terms of ADL and iADL) were significantly different among the cognitive subgroups. Activity as measured on Frenchay Activity Index decreased with increasing cognitive impairment (28.1 ± 6.9, 21.6 ± 6.6, 13.8 ± 6.9 respectively), as did hand grip strength (22.7 ± 8.2, 18.3 ± 6.8, 16.3 ± 5.9 respectively) and SPPB performance (9.7 ± 2.3, 8.9 ± 2.0), 6.5 ± 2.4 respectively) (all *p* < 0.05) (see Table 1).

### Comparison between frailty state transitions and longitudinal cognitive performance (Fig. 1)

**Fig. 1:**
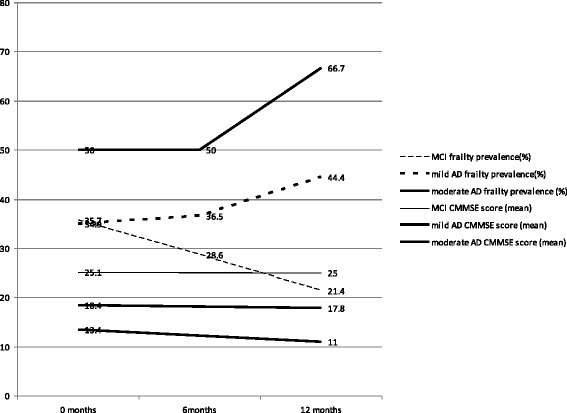
Frailty prevalence at baseline, 6 months and 12 months and cognitive scores at baseline and 12 months

In the overall group, 41.0 % were frail at baseline, 37.6 % frail at 6 months and 43.4 % frail at 12 months. The prevalence of frailty in MCI, mild and moderate AD cognitive subgroups to vary between the 6-month intervals (presented as proportions; 0.36 (95 % confidence interval (CI) (0.15–0.64) vs 0.35 (95 % CI 0.24–0.48) vs 0.50 (95 % CI 0.23–0.77) at baseline; 0.29 (95 % CI 0.11–0.58) vs 0.37 (95 % CI 0.25–0.49) vs 0.50 (95 % CI 0.23–0.77) at 6 months and 0.21 (95 % CI 0.07–0.51) vs 0.44 (95 % CI 0.32–0.57) vs 0.67 (95 % CI 0.36–0.88) at 12 months for MCI, mild and moderate AD respectively) while CMMSE scores for MCI, mild and moderate AD follow a predictable linear pattern of cognitive decline (25.2 ± 3.4 vs 18.4 ± 4.6 vs 13.3 ± 5.4 at baseline, and 25.0 ± 2.4 vs 17.8 ± 5.1 vs 11.0 ± 6.3 at 12-month for MCI, mild and moderate AD respectively) [30].

### Frailty status progression

**Fig. 2:**
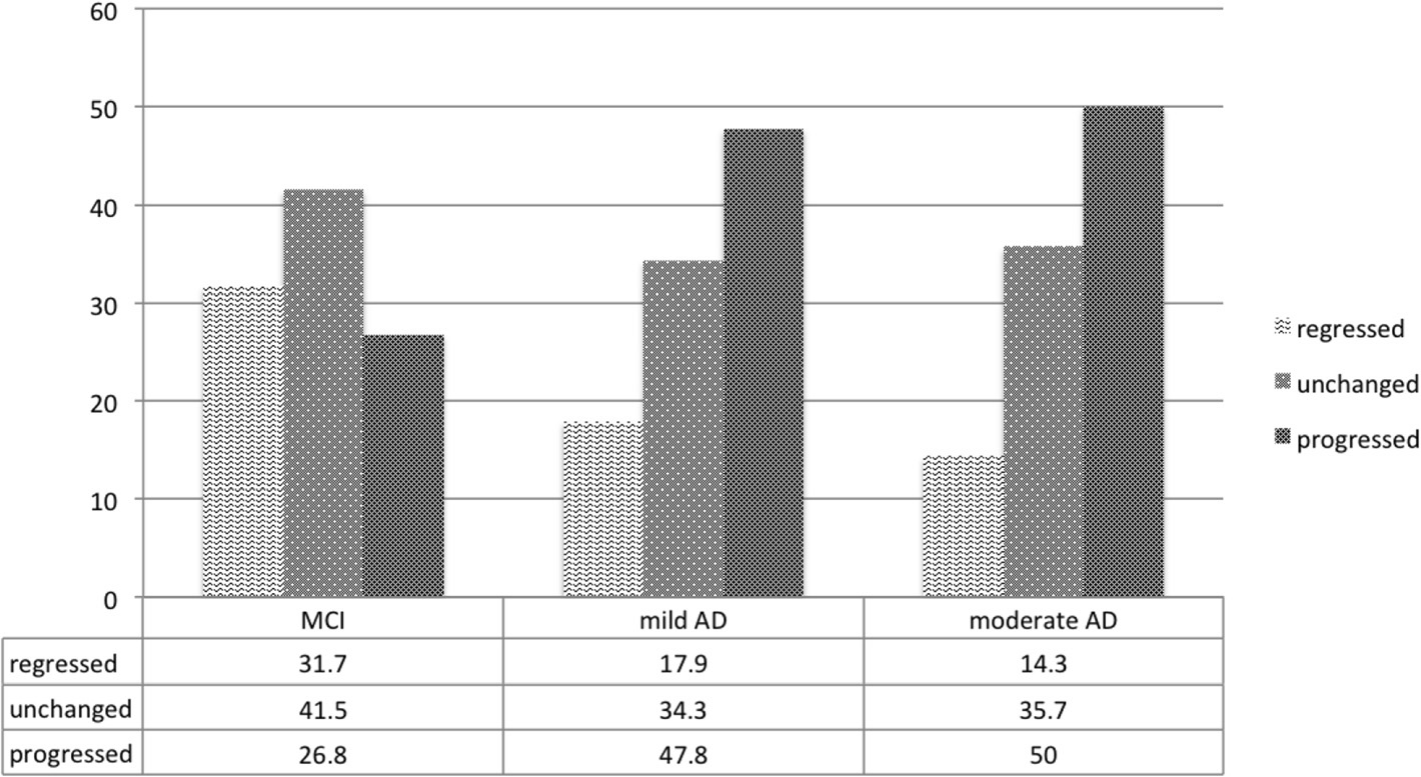
Percentage of frailty transition status in the 3 cognitive subgroups (MCI, mild and moderate AD)

As a whole group, 39 subjects (32 %) had frailty regression, 44 (36 %) remained unchanged while 39 (32 %) progressed during the one-year follow-up. Notably, among cognitive subgroups, the majority of MCI subjects either regressed (31.7 %) or remained unchanged (41.5 %) in frailty status, whereas mild and moderate AD tended to progress (47.8 and 50.0 % respectively) although these observed differences did not reach statistical significance on chi-squared test (*p* = 0.20) (Fig. 2).

### Comparison of patient factors between 3 frailty transition status (Table 2)

**Table 2:** Comparison of baseline patient factors between 3 frailty transition states (*n* = 122)

	Total (n-122)	Regressed (*n* = 39)	Unchanged (*n* = 44)	Progressed(*n* = 39)
Demographics				
Age	75.4 (7.1)	77.1 (6.9)	73.9 (7.2)	75.3 (7.1)
Gender (Male %)	40.1	38.5	36.4	46.2
Race (Chinese %)	94.3	92.3	97.7	92.3
Education years	7.1 (5.0)	6.6 (4.8)	7.3 (4.5)	7.3 (5.8)
CDR global	0.9 (0.5)	0.9 (0.5)	0.9 (0.4)	0.9 (0.5)
CDR Sum of boxes	4.0 (3.0)	4.6 (2.9)	3.8 (2.7)	3.6 (3.4)
CMMSE	20.0 (6.5)	19.2 (5.3)	20.9 (5.2)	20.1 (6.5)
Medical comorbidities				
HT (%)	65.6	74.4	54.6	69.2
DM (%)	29.5	38.5	20.5	30.8
Hyperlipidemia (%)	70.5	66.7	70.5	74.4
IHD (%)	21.3	23.1	25.0	15.4
Smoker/ex-heavy smoker (%)	15.5	12.8	18.2	15.3 *
AF (%)	3.3	5.1	0.0	5.1
PVD (%)	0.8	2.6	0	0
Multimorbidity score	2.1 (1.2)	2.2 (1.2)	1.9 (1.2)	2.1 (1.3)
MDD (%)	2.5	2.6	2.3	2.6
Neurodegeneration and wm scores				
APOE status				
E2/e2	0.8	2.6	0	0
E2/e3	0.2	0.1	0.1	0
E2/e4	1.6	2.6	2.3	0
E3/e3	47.5	43.6	47.7	51.3
E3/e4	32.8	33.3	31.8	33.3
E4/e4	4.9	7.7	4.6	2.6
MTA score	1.2 (1.1)	1.2 (1.1)	1.3 (1.0)	1.2 (1.1)
ARWMC score	4.9 (4.2)	5.0 (5.2)	5.5 (4.1)	4.2 (3.1)
Function, behaviour and burden scores				
ADL	97.8 (5.8)	95.9 (9.0)	99.0 (2.8)	98.5 (3.1)*^a^
iADL	15.7 (13.7)	13.7 (5.9)	16.5 (5.1)	16.8 (4.6) *^a, b^
NPI total severity	2.1 (1.7)	2.2 (1.8)	2.3 (1.9)	1.7 (1.3)
NPI CG distress	3.6 (5.3)	3.9 (6.0)	4.4 (5.5)	2.2 (3.9)
Zarit total	22.6 (16.1)	21.6 (12.6)	24.1 (19.8)	21.9 (14.7)
FAI total	22.9 (8.1)	21.0 (7.9)	24.9 (8.7)	22.7 (7.1)
Nutrition, lean mass and functional performance				
MNA total	11.8 (1.4)	11.6 (1.2)	118 (1.6)	12.2 (1.4)
Grip strength	19.5 (7.5)	18.1 (7.3)	20.6 (8.0)	19.8 (7.1)
SPPB total	8.7 (2.3)	8.6 (2.1)	9.2 (2.2)	8.2 (2.6)
Lean mass measurement	6.6 (8.2)	8.0 (13.9)	5.9 (0.9)	5.8 (0.9)
Treatment				
Donepezil	44.5	42.1	41.9	50.0
Galantamine	0	0	0	0
Rivastigmine	4.2	5.3	4.7	2.6
Memantine	22.7	34.2	25.6	7.9 *
Socioeconomic and lifestyle factors				
Expenses				
More than enough	34.4	60.1	44.4	63.0
Fair	37.5	33.3	44.4	29.6
Not enough	28.1	3.0	11.1	7.4
Lifestyle factors (%)				
Daily Activities				
1 + h	33.3	39.4	22.2	40.7
20-59 min	31.3	27.3	38.9	25.9
< 20 min	19.8	18.2	22.2	18.5
none	15.6	15.2	16.7	14.8
Vegetable and fruit intake (%)				
Daily	83.3	78.8	86.1	85.2
< Daily	16.7	21.2	13.9	14.8
Fish intake (%)				
Daily	41.7	30.3	55.6	37.0
< Daily	58.3	69.7	44.4	63.0 (*p* = 0.08)
Social support factors (%)				
Neighbours				
None	22.9	27.3	22.2	18.5
1-4	49.0	45.4	52.8	48.2
5-9	14.6	12.1	11.1	22.2
10+	13.5	15.2	13.9	11.1
Laboratory investigations				
Total chol	4.9 (1.1)	5.1 (1.1)	4.8 (1.2)	5.0 (0.9)
LDL	2.8 (0.9)	3.0 (1.0)	2.6 (0.9)	2.8 (0.8)
HDL	1.6 (0.7)	1.6 (0.5)	1.6 (0.7)	1.7 (0.7) *
TG	1.3 (0.7)	1.2 (0.7)	1.4 (0.9)	1.2 (0.5) *
Vit D level	30.3 (11.9)	29.5 (13.1)	29.6 (10.6)	32.1 (12.1)

*Abbreviations*: *CDR* clinical dementia rating, *CMMSE* Chinese mini mental state examination (range 0–28), *HT* hypertension, *DM* diabetes mellitus, *IHD* ischaemic heart disease, *AF* atrial fibrillation, *PVD* peripheral vascular disease, *BMI* body-mass index, *ETOH* ethanol, *MDD* diagnostic statistical manual definition of major depressive disorder, *MTA* medial temporal atrophy, *ARWMC* age-related white matter changes, *ADL* activities of daily living (range 0–100), *CG* caregiver; activities of daily living (range 0–23), *NPI* neuropsychiatric inventory, *FAI* frenchay activity index, *MNA* mini-nutritional assessment, *SPPB* short physical performance battery, *LDL* low-density lipoprotein, *HDL* high-density lipoprotein, *TG* triglyceride, *Vit D* Vitamin D

*p < 0.05 between the 3 frailty transition subgroups (ANOVA with Bonferroni correction for parametric and Kruskal-Wallis test for nonparametric continuous variables; Chi-square test for categorical variables)

^a^differences between frailty regressed and unchanged state

^b^differences between frailty regressed and progressed state

Baseline cognitive status on CMMSE and CDR-SB were not significantly different in the 3 frailty transition groups, nor did baseline measures of neurodegeneration and white matter lesions on MTA and ARWMC. Significant univariate differences were noted in smoking status, functional status (ADL and iADL), and laboratory measurements of HDL and TG (all *P* < 0.05), whilst daily fish intake (*p* = 0.08) approached significance (Table 2).

### Random effects modelling with longitudinal frailty score as the outcome variable

**Fig. 3:**
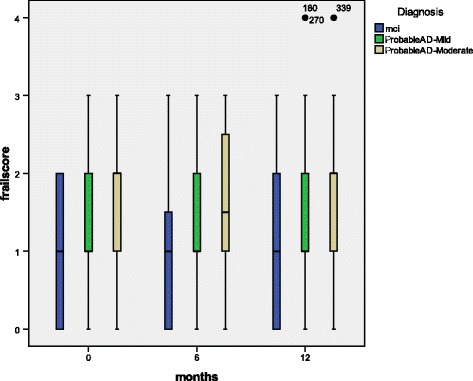
Frailty score at baseline, 6 month and 12 month for 3 cognitive subgroups (*n* = 122)

We subsequently looked at longitudinal frailty scores via the 3 cognitive subgroups at baseline, 6 months and 12 months (Fig. 3). We performed random effects modelling with independent variables of age, gender, smoking history, functional status (iADL), lifestyle factors (fish intake) and biochemical parameters (HDL and TG levels) and longitudinal CDR-SB performance for the whole group. Age (coeff 0.09, 95 % CI: 0.04–0.15, *p* = 0.001) and cognition (CDR-SB) (coeff 0.04, 95 % CI: 0.02–0.07, *p* = 0.000) were significantly associated with frailty score (Table 3). When we performed random effects modelling only on MCI subjects (*n* = 41), only female gender was significantly associated with frailty score (coeff 1.28, 95 % CI 0.21–2.36, *p* = 0.019) (Table 4). Subsequent analyses with mild-moderate AD subjects (*n* = 81 showed similar findings to whole group analyses where age (coeff 0.04, 95 % CI 0.02–0.07, *p* = 0.001) and cognition (coeff 0.11, 95 % CI 0.05–0.17, *p* = 0.000) remained significantly associated with frailty score (Table 5).

**Table 3:** The associations of the frailty score with the various factors using the random effect model (whole group *n* = 122)

	Coeff	Std Err	*p*-value	95 % Confidence interval
Frailty score				
Age	0.04	0.012	0.000	0.02–0.07
Gender	0.22	0.18	0.224	−0.13–0.58
Smoking	−0.01	0.23	0.964	−0.47–0.45
iADL	−0.004	0.02	0.80	−0.04–0.03
Fish intake	−0.03	0.15	0.84	−0.32–0.26
HDL	−0.01	0.13	0.93	−0.27–0.24
TG	0.02	0.11	0.82	−0.19–0.24
CDR-SB	0.09	0.03	0.001	0.04–0.15

*Abbreviations*: *Std Err* standard error, *iADL* independent activities of daily living, *HDL* high-density lipoprotein, *TG* triglyceride, *CDR-SB* clinical dementia rating sum-of-boxes score

**Table 4:** The associations of the frailty score with the various factors using the random effect model (MCI subjects *n* = 41)

	Coeff	Std Err	*p*-value	95 % Confidence interval
Frailty score				
Age	0.04	0.06	0.49	−0.07–0.16
Gender	1.28	0.55	0.02	0.21–2.36
Smoking	0.07	0.78	0.94	−1.50–1.62
iADL	−0.10	0.08	0.22	−0.26–0.06
Fish intake	0.04	0.53	0.94	−0.99–1.08
HDL	−0.05	0.30	0.86	−0.63–0.53
TG	0.33	0.54	0.55	−0.74–1.39
CDR-SB	−0.31	0.39	0.43	−1.08–0.46

*Abbreviations*: *Std Err* standard error, *iADL* independent activities of daily living, *HDL* high-density lipoprotein, *TG* triglyceride, *CDR-SB* clinical dementia rating sum-of-boxes score

**Table 5:** The associations of the frailty score with the various factors using the random effect model (mild-moderate AD subjects *n* = 81)

	Coeff	Std Err	*p*-value	95 % Confidence interval
Frailty score				
Age	0.04	0.01	0.001	0.02–0.07
Gender	0.15	0.20	0.47	−0.25–0.54
Smoking	0.06	0.27	0.83	−0.46–0.58
iADL	0.006	0.02	0.76	−0.03–0.04
Fish intake	−0.11	0.16	0.49	−0.42–0.20
HDL	−0.15	0.18	0.42	−0.50–0.21
TG	0.01	0.11	0.94	−0.21–0.22
CDR-SB	0.11	0.03	0.000	0.05–0.17

*Abbreviations*: *AD*: Alzheimer’s dementia, *Std Err* standard error, *iADL* independent activities of daily living, *HDL* high-density lipoprotein, *TG* triglyceride, *CDR-SB* clinical dementia rating sum-of-boxes score

We performed subgroup analyses on subgroup with cognitive frailty (*n* = 18) and showed that those who progressed had more comorbidities (diabetes (4.0 compared to 1.7 % unchanged and 0 % regressed); ischaemic heart disease (33.3 compared to 66.7 % unchanged and 0 % regressed)), less family support (children) (40 compared to 60 % regressed), were functionally more impaired (in terms of iADL) (12.3 ± 6.1, 20.4 ± 2.4 unchanged and 18.7 ± 3.7 regressed ), had poorer performance on chair stand test (1.0 ± 0, 4.0 ± 0 unchanged compared to 2.7 ± 0.6 regressed) with paradoxically larger lean muscle mass (6.8 ± 0.3 kg/m2 progressed, 5.1 ± 0 kg/m2 unchanged compared to 5.3 ± 0.4 kg/m2 regressed) (all *p* < 0.05).

## Discussion

The frailty syndrome has attracted attention as a potential therapeutic target due to its relation both as a precursor and contributor of disability in older persons. More recently, experts have focused on cognitive frailty [4, 5]. This is the first study to demonstrate frailty state transitions over a one-year period in subjects with cognitive impairment. It is important to note that these subjects were near full independence in their basic ADLs (Table 1), indicating that they were a cohort of relatively well older adults in whom potential intervention could result in reversibility of their conditions.

Contrary to expectations of increasing frailty with increasing cognitive impairment, our previous published work showed the U-shaped relationship between frailty and increasing cognitive impairment, characterised by the initial dissociation with cognitive impairment and subsequent convergence at later stages [16]. These interesting findings have been discussed in detail in the earlier paper where these apparently contradictory differences could be partially explained, or whether a conceptual or measurement issue exists for the observed differences in frailty prevalence. These observations require a larger sample size to confirm these conclusions. Additionally, we suggested further research in the fatigue sub-item in the biological frailty model to see if the fatigue sub-item is driven by physical or psychological components [16]. Our current results demonstrate consistent results longitudinally. This is seen in the MCI stage where longitudinal frailty status and scores now show a linear decline over 12-month follow up, along with almost one-third with frailty regression, which supports the potential reversibility of cognitive frailty. These findings are further supported by random effects modelling results showing that in mild-moderate AD subjects, cognitive changes (as shown by CDR-SB changes) and age are directly associated with frailty progression while in MCI subjects, gender and potentially other mechanisms (unmeasured in this current study), may influence physical frailty progression. Contrary to population study findings, frailty progression in patients with established cognitive impairment appears to be associated with age and cognition, which supports the need for cognitive therapies in those already with diagnosed dementia. These initial findings also support cognitive frailty as a significant and potentially modifiable entity whereby frailty interventions may have a role in preventing physical and functional decline in cognitively impaired older adults. Our study showed differential findings to other community epidemiological studies [12] where female gender was less likely to result in decline in frailty status compared to men in MCI subjects (Table 4).

MCI remains a heterogeneous clinical construct with different underlying pathological processes (neurodegeneration, vascular, mood, metabolic) when they present in the clinical setting. In our study, we have shown that 16.7 % subjects with cognitive frailty (both MCI and physical frailty) had frailty progression after one year. The analyses of this small subgroup (*n* = 18) showed that those who progressed had more comorbidities (diabetes, ischaemic heart disease), less family support (children), were functionally more impaired (in terms of iADL), had poorer performance on chair stand test with paradoxically larger lean muscle mass (results not shown). This seemed independent of the APOEe4 status or neuro-degeneration measures of medial temporal atrophy and ischaemic vascular lesions, which potentially add to further insights into the differentiation of a physically driven process, compared to a purely cognitive (neurodegeneration) driven process [5, 39, 40]. These interesting findings (despite a small sample size), requires further validation in future carefully clinically-phenotyped subjects in larger MCI studies.

The strengths of our study include the comprehensive data available on both cognition and frailty measures. However, we were limited by the relatively small sample size and short follow-up period of one year. Other than the current conventional laboratory measurements, we had not included other biomarker measurements (such as inflammatory and hormonal markers) in the current paper, which might provide insights into the biological underpinnings and further understanding of the complex relationship between frailty and cognition [5]. Additionally, a matched control sample of healthy subjects without cognitive impairment could have aided in showing cognitive frailty is a separate entity, driving frailty progression. A separate analyses (albeit a different methodology) has suggested a concomitant pro-inflammatory state without concomitant endocrine deficiency, to adversely influence baseline and progressive physical frailty [41].

## Conclusion

In summary, this is the first study to demonstrate longitudinal frailty state transitions in cognitively-impaired older adults. Contrary to expectations of frailty progression to be closely correlated with increasing cognitive impairment, they appear independent, especially in the earliest stage of cognitive impairment. This study lends initial support to ‘cognitive frailty’ entity being a potentially modifiable factor for physical frailty progression. It further allowed evidence for potential refinement of this clinical construct for development of an ‘at-risk’ cognitive frailty phenotype. Further research is needed with a suitably powered study over a longer follow-up period with additional focus on pathogenic mechanisms to allow rapid translation of knowledge into useful therapeutic interventions.

## Abbreviations

MCI: mild cognitive impairment
AD: Alzheimer’s disease
CDR-SB: clinical dementia rating- sum of boxes
DSRB: domain specific review board
NHG: National Healthcare Group
CMMSE: Chinese Mini Mental State Examination
NINCDS-ADRDA: National Institute of Neurological and Communicative Disorders and Stroke and Alzheimer’s Disease and Related Disorders Association
CDR: clinical dementia rating
ADL: activities of daily living
iADL: independent activities of daily living
DSM-IV: diagnostic and statistical manual
CT: computed tomography
WML: white matter lesions
ARWMC: age-related white matter changes
MTA: medical temporal atrophy
MNA: mini nutritional assessment
DXA: dual energy x-ray absorptiometry
SPPB: short physical performance battery
HDL: high-density lipoprotein
LDL: low-density lipoprotein
APOEε4: apolipoprotein E4
